# Circulating Omega‐3 Fatty Acids and Cardiometabolic Outcomes: A Systematic Review of Publications From UK Biobank

**DOI:** 10.1002/lipd.70076

**Published:** 2026-07-29

**Authors:** Warda Tul Firdous, Philip C. Calder

**Affiliations:** ^1^ School of Human Development and Health, Faculty of Medicine University of Southampton Southampton UK; ^2^ NIHR Southampton Biomedical Research Centre, University Hospital Southampton NHS Foundation Trust and University of Southampton Southampton UK

**Keywords:** cardiovascular disease, diabetes, docosahexaenoic acid, Eicosapentaenoic acid, heart disease, liver disease

## Abstract

Studies have shown that omega‐3 fatty acids may protect against cardiovascular diseases (CVDs) and related metabolic conditions. UK Biobank is a large cohort study that includes data on plasma omega‐3 fatty acids at study entry and health outcomes during the follow‐up period. The aim of this research is to explore the association between plasma omega‐3 fatty acids (total omega‐3 fatty acids, docosahexaenoic acid (DHA) and non‐DHA omega‐3 fatty acids) and cardiometabolic health outcomes by systematically reviewing publications from UK Biobank. Three databases (EMBASE, MEDLINE, CINAHL) were searched in September 2025 using key words. Based on inclusion/exclusion criteria, 16 publications were selected for inclusion (two cross‐sectional and 14 longitudinal with a follow‐up of 11 to 14 years). Sample size varied between 20,338 and 273,834 across the included studies. Eleven studies reported on CVD outcomes and five on metabolic outcomes. Plasma total omega‐3 fatty acids and DHA were inversely associated with risk of coronary heart disease, CVD mortality, heart failure, haemorrhagic stroke and severe fatty liver disease. Plasma total omega‐3 fatty acids were also inversely associated with risk of prediabetes and type‐2 diabetes, while plasma DHA was also inversely associated with risk of coronary heart disease mortality, ischemic stroke and fatty liver disease. It is concluded that publications based on UK Biobank consistently demonstrate a significant association between higher levels of total omega‐3 fatty acids or DHA in plasma and lower likelihood of poor cardiometabolic health outcomes, providing support for guidance for the population to achieve recommended intakes of these fatty acids.

**Abbreviations**
CHD: coronary heart disease
CI: confidence interval
CVD: cardiovascular disease
DHA: docosahexaenoic acid
EPA: eicosapentaenoic acid
HR: hazard ratio
MASLD: metabolic‐associated steatotic liver disease
NAFLD: non‐alcoholic fatty liver disease
NMR: nuclear magnetic resonance
OR: odds ratio
T2D: type‐2 diabetes
UKBB: UK Biobank

## 1 Introduction

Cardiovascular diseases (CVDs) are a global health burden, being the major cause of morbidity and mortality worldwide (Mensah et al. 2023); they account for over 30% of all global deaths, estimated at 18 million per year. Although there are numerous types of CVDs (e.g., coronary heart disease (CHD), peripheral artery disease, cerebral artery disease) and numerous manifestations (e.g., myocardial infarction, heart failure, ischaemic stroke), CVDs share common risk factors which contribute to or exacerbate the disease, such as age, sex, genetics, hypertension, family history, increased cholesterol, other dyslipidaemias, inflammation, obesity, smoking, and uncontrolled diabetes (Adhikary et al. 2022). Metabolic diseases, such as type‐2 diabetes mellitus (T2D) and liver diseases like metabolic‐associated steatotic liver disease (MASLD), previously called non‐alcoholic liver fatty disease (NAFLD), share common risk factors with CVDs, such as impaired glucose metabolism, hypertension and obesity (Silveira Rossi et al. 2021), and such metabolic diseases can increase the likelihood of CVDs (Tune et al. 2017; Targher et al. 2024). Risk of metabolic and cardiovascular diseases is controlled through healthy lifestyle, including healthy eating, and use of medications that target risk factors such as hypertension, high cholesterol and insulin resistance (Lichtenstein et al. 2026; Netala et al. 2024).

The Global Burden of Disease Study 2017 reported that a diet “low in seafood omega‐3 fatty acids” was the sixth leading dietary risk factor for mortality and morbidity globally, especially due to CVDs (GBD 2017 Diet Collaborators 2019). Seafood omega‐3 fatty acids include two key fatty acids eicosapentaenoic acid (EPA) and docosahexaenoic acid (DHA), which play a crucial role in the body (Calder 2018; Swanson et al. 2012), especially in promoting cardiometabolic health (Djuricic and Calder 2023, 2025). Found primarily in marine‐derived sources, such as oily fish and algae (Lewis et al. 2025) and supplements based on fish, krill or algal oils, EPA and DHA serve vital biological and cellular roles and in the context of CVDs act to lower triglycerides (Harris 2001), control inflammation (Calder 2020), promote vascular function (Wang et al. 2012) and support cardiac function (Hidayat et al. 2018) as well as having modest hypotensive effects (Miller et al. 2014). There is a long history of research on EPA and DHA and risk and treatment of CVDs, especially CHD, as reviewed elsewhere (Djuricic and Calder 2023, 2025; Innes and Calder 2020). There is good evidence from cohort studies that higher intake or blood levels of EPA and DHA are associated with lower risk of CVDs (Alexander et al. 2017; Chowdhury et al. 2014; Del Gobbo et al. 2016; Harris et al. 2021) and metabolic conditions such as T2D (Qian et al. 2021). This evidence underpins recommendations for intake of EPA and DHA to promote and maintain health, especially cardiovascular health (Calder et al. 2025). Support for benefits of EPA and DHA also comes from randomized controlled trails examining their effects on risk factors (AbuMweis et al. 2018; Choi and Calder 2024) and on cardiovascular outcomes, where multiple meta‐analyses over the last years report reductions in cardiovascular events or mortality (Bernasconi et al. 2021; Dinu et al. 2024; Hu et al. 2019; Huang et al. 2023; Khan et al. 2021; Xie et al. 2021). Nevertheless, there are null trials (ASCEND Study Collaboration Group 2018; Kalstad et al. 2021; Nicholls et al. 2020; ORIGIN Trial Investigators 2012) and meta‐analyses (Rizos et al. 2021) of EPA and DHA and cardiovascular outcomes; inconsistencies around dose and differential effects of EPA and DHA have been highlighted (Sherratt et al. 2023; Toth et al. 2022); and some have argued that preformed EPA and DHA are not actually needed (Burdge 2022). In the absence of trials investigating primary prevention of cardiometabolic disease with EPA and DHA, cohort studies (and their pooling) provide the main evidence for their association with disease incidence. In the light of the continued discussion about a role for EPA and DHA in primary prevention, there is a need to continue to examine the relationships between EPA and DHA intake and biomarker levels and incidence and progression of cardiometabolic disease.

Advantages of cohort studies often include their large size and long duration of follow‐up. With regard to EPA and DHA, one disadvantage is that they often rely on dietary intake data which are difficult to obtain accurately due to limited and irregular intake of the main food sources (fatty fish) and poor food composition tables. Information on omega‐3 biomarkers such as levels in blood or blood cells is advantageous and some cohort studies have these data for all or some participants. Nevertheless, comparing or combining data across several cohorts has limitations because of differences in age and sex distribution, ethnicity, background diet, follow‐up time, outcome definition, biomarker analysis methodology and so on. UK Biobank (UKBB) with its detailed phenotyping and multiple outcomes reported in the same population offers an opportunity to explore the relationships between blood omega‐3 fatty acids and cardiometabolic outcomes within a large, well‐defined and reasonably heterogeneous cohort.

Details of the UKBB cohort can be found elsewhere (Sudlow et al. 2015). The UKBB recruited 503,317 participants aged 40 and 69 years between 2006 and 2010; approximately 9.2 million UK residents aged 40 to 69 years were invited to participate. Participants lived in different parts of the UK but within a 25‐mile radius of a study center; about 86% of participants lived in an urban area at the time of recruitment. More females than males were recruited (54% vs. 46%); 24% of recruits were aged 40 to 49 years, 34% 50 to 59 years and 42% 60 to 69 years. Participants were mostly (94.6%) of white ethnicity, which reflected the demographic at the time of recruitment (94.5% white in 2001 and 91.3% in 2011); around 8% of recruits were born outside of the UK. UKBB participants were on average slightly wealthier and healthier than the UK population at the time of recruitment. UKBB participants continue to be followed over time including collecting data on a number of health‐related outcomes such as incidence of CHD, CVDs and related events (e.g., heart failure) and risk of T2D, MASLD and so on. In UKBB plasma omega‐3 fatty acids were measured using nuclear magnetic resonance (NMR) spectroscopy, and the reported data included the total omega‐3 fatty acids, DHA and non‐DHA omega‐3 fatty acids. The aim of this research is to investigate if there is any association between circulating omega‐3 fatty acids and cardiometabolic health outcomes, through a systematic review of studies published using data from the UKBB.

## 2 Methods

### 2.1 Literature Searching

This systematic review was conducted in accordance with the guidelines of Preferred Reporting for Systematic Reviews and Meta‐Analyses (PRISMA; Page et al. 2021). It was registered as “Association of circulating omega‐3 fatty acids and cardiometabolic outcomes: a systematic review of publications from UK Biobank” at the International Platform of Registered Systematic Review and Meta‐analysis Protocols (INPLASY) as protocol INPLASY202610041.

Three electronic databases (EMBASE, MEDLINE, and CINAHL) were searched for relevant studies that addressed the research question and were consistent with the PEO framework:

- Population: UK Biobank participants.
- Exposure: Omega‐3 fatty acids, such as EPA and DHA, in circulating blood.
- Outcome(s):
  ○ Incidence of cardiometabolic disease, such as, but not limited to, CVD, CHD, heart failure, myocardial infarction, stroke, hypertension, atrial fibrillation, T2D, and NAFLD/MASLD;
  ○ Mortality from cardiometabolic disease, such as, but not limited to, CVD, CHD, myocardial infarction, stroke, and NAFLD/MASLD.

The three databases were searched in September 2025 using keywords and their alternative terms. The search terms were ((omega 3 OR omega‐3) OR (PUFAs OR polyunsaturated fatty acids) OR (EPA OR eicosapentaenoic acid) OR (DHA OR docosahexaenoic acid)) AND (UK Biobank OR UK Bio Bank OR UKBB). The search string is shown in Table S1.

### 2.2 Selection of Publications for Inclusion in the Systematic Review

The identified publications were entered into EndNote 21, where they were deduplicated and then exported to a Microsoft Excel spreadsheet. Publications were initially screened using pre‐established inclusion and exclusion criteria by reviewing the titles and abstracts. The inclusion criteria were: reported a clinically relevant outcome, reported omega‐3 fatty acid concentrations in plasma or blood, were available as full‐text articles, and were published in the English language. Publications that were excluded reported dietary intake of omega‐3 fatty acids only or failed to report relevant clinical outcomes. Furthermore, studies that utilized Mendelian randomization only were also excluded. Remaining publications were assessed for eligibility by accessing and reviewing the full texts.

### 2.3 Quality Assessment

The quality of each included prospective cohort study was assessed using the Newcastle‐Ottawa Quality Assessment Scale for Cohort Studies (Wells et al. 2000). The higher the score, the higher the study quality. There is a maximum score of 9 spread across three domains: selection (maximum score of 4), comparability (maximum score of 2), and outcome (maximum score of 3).

The quality of the two cross‐sectional studies was assessed using a modified Newcastle‐Ottawa Quality Assessment Scale (Carra et al. 2026). The higher the score, the higher the study quality. There is a maximum score of 9 spread across three domains: study sample selection (maximum score of 2), assessment of exposure and outcome (maximum score of 4), and confounding factors (maximum score of 3).

## 3 Results

### 3.1 Selection of Publications for Inclusion

Three electronic databases (EMBASE, MEDLINE, and CINAHL) were searched for relevant publications. Respectively, 235, 139, and 32 publications were identified, totalling 406. After de‐duplication, 284 publications remained. These were screened by title and abstract to establish whether they were relevant to the systematic review, according to the inclusion and exclusion criteria (see Methods). After screening, 23 publications were retrieved and analyzed for eligibility by full‐text searching; however, 7 publications were not included (Figure 1). One of those 7 publications one combined data from UKBB with data from two other cohort studies (Shi et al. 2025), one related plasma omega‐3 fatty acids to dietary patterns (Liu, Liu, et al. 2025), one was about omega‐3 supplement use (Vell et al. 2023), and four did not report relevant clinical outcomes (Guo et al. 2024; Kelly et al. 2021; Liu et al. 2024; Wang et al. 2025). Therefore, 16 publications/studies were included (Figure 1). These all reported plasma omega‐3 fatty acid data from NMR analysis expressed as total omega‐3 PUFAs, DHA, and non‐DHA omega‐3 PUFAs.

**FIGURE 1:**
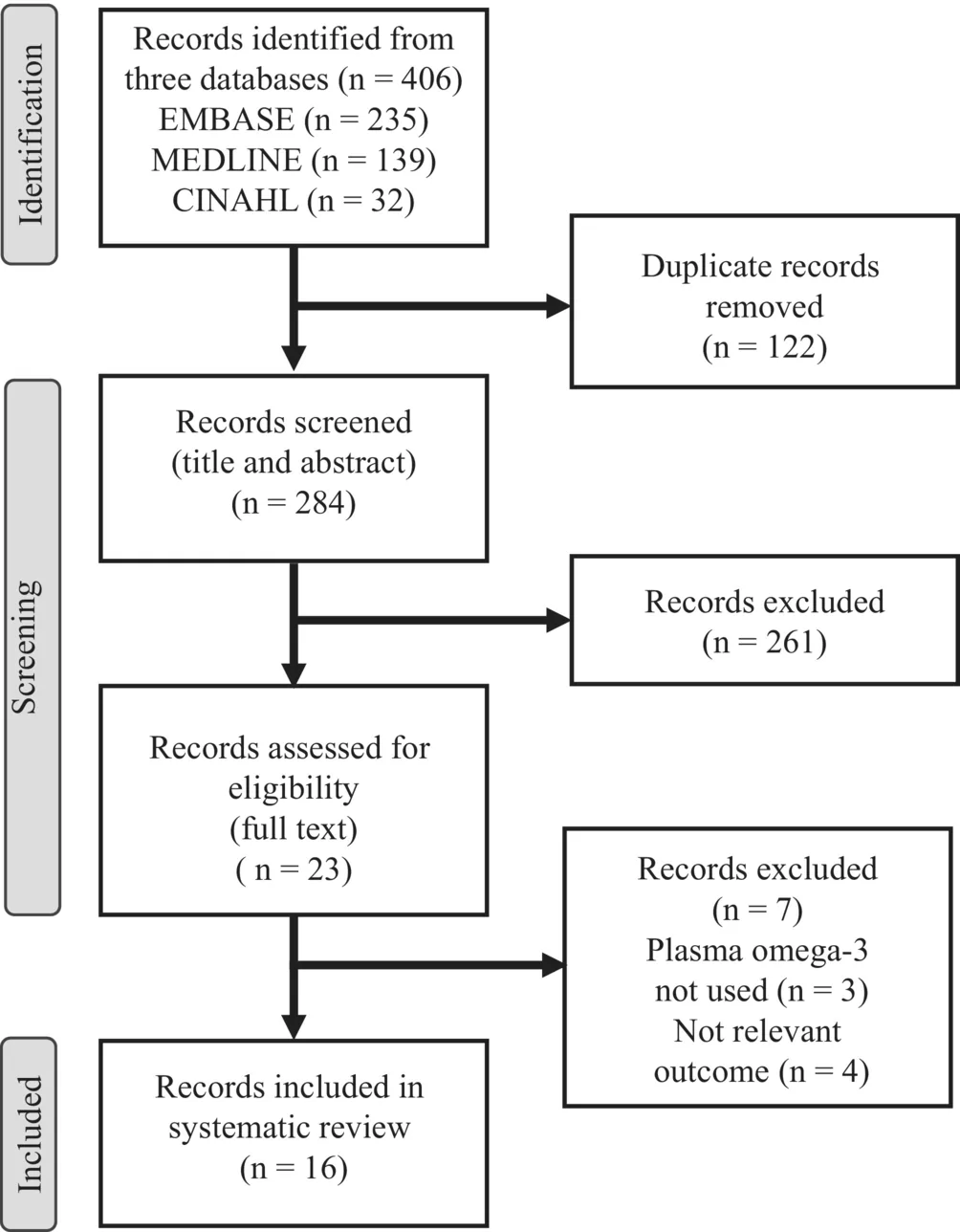
PRISMA diagram showing selection of studies for inclusion.

### 3.2 Characteristics of the Included Studies

Table 1 presents the characteristics of the 16 included studies, summarized according to the population, participant characteristics, study size, duration of follow‐up, and clinical outcomes reported. The cardiometabolic outcomes that were examined comprised incidence of CHD, CHD mortality, CVD mortality, heart failure, hypertension, prediabetes, T2D, and NAFLD/MASLD. Sample size varied between 20,338 and 273,834 across the reported studies. Of the 16 studies, two were cross‐sectional (Schuchardt et al. 2024; Tian et al. 2023); the others were longitudinal with a follow‐up of 11–14 years.

**TABLE 1:** Characteristics of studies included in the systematic review.

Study	Population	Sample size (*n*)	Duration of follow‐up	Cardiometabolic outcomes reported
Jin et al. (2023)	Mean age: 55.2 ± 8.0 years Male: 42.8% Mean body mass index: 27.0 ± 4.6 kg/m^2^ White: 95% Excluded participants with CHD, aged less than 40 or more than 70 years at baseline, who withdrew, were missing fatty acid biomarker data or covariates, who had outlying values of fatty acids, or were taking lipid‐lowering medication, like statins	89,242	Median 11.8 years	Incident CHD: Defined as the first‐ever myocardial infarction or unstable angina pectoris, coronary‐related death, or coronary‐related procedures (coronary artery bypass surgery or percutaneous transluminal angioplasty stent placement)
Zhuang et al. (2023)	Mean age: ~55.9 ± 8.0 years Male: 45.2% Mean body mass index: ~27.8 ± 4.7 kg/m^2^ White: 94% Excluded participants who withdrew from the study, or who had a history of CVD or cancer	101,367	Mean 11.5 years	Incident CHD: Defined as myocardial infarction, coronary‐related death or coronary‐related procedures (coronary artery bypass or triple heart bypass surgery, coronary angioplasty with or without stent placement)
Fan et al. (2023)	Mean age: 55.4 ± 8.1 years Male: 47.9% Mean body mass index: 27.2 ± 4.7 kg/m^2^ White: 95% Selection criteria for participants not provided	74,713	Mean 14 years	CHD mortality
Zhang et al. (2024)	Mean age: ~55.9 ± 8.0 years Male: 47% Mean body mass index: ~27.2 ± 4.6 kg/m^2^ White: 91% Excluded participants with cancer (*n* = 37,736) or CVD (*n* = 100,972), who withdrew from the study (*n* = 879), or who had incomplete data on the plasma omega‐6/omega‐3 ratio (*n* = 277,372)	85,425	Mean 12.7 years	CVD mortality
O'Keefe et al. (2024)	Mean age: 57 years Male: 46% Mean body mass index: 27.0 kg/m^2^ White: 94% Excluded participants who died within one year of follow‐up	117,702	Median 12.7 years	CVD mortality
Zhang et al. (2025)	Median age: 58 (50–63) years Male: 45.4% White: 95% Excluded participants with any type of major adverse cardiovascular event or those with missing biomarker data at baseline	229,352	13.66 years	New onset major adverse cardiovascular events defined as incident myocardial infarction, incident stroke (ischemic or haemorrhagic), death from CVD, or the occurrence of unstable angina
Ren et al. (2025)	Median age: 51 (45–55) years Male: 44.9% Median body mass index: 26.2 (23.7–29.3) kg/m^2^ White: 94% Excluded participants with a previous diagnosis of CHD, cerebrovascular disease, heart failure or cardiac arrest, on lipid‐lowering medication, or with missing covariates	54,561	Median 12.3 years	Incidence of major adverse cardiovascular events defined as CHD, stroke or CVD mortality
Liu, Ao, et al. (2025)	Mean age: ~55.5 ± 8.0 years Male: 45.9% Mean body mass index: ~27.4 ± 4.7 kg/m^2^ White: 94% Excluded participants without a complete fatty acid profile, lost to follow‐up, with CVD or cancer, or who died within the first 5 years of follow‐up	117,871	Median 11.9 years	CVD mortality
Ma et al. (2023)	Mean age: 56.5 ± 810 years Male: 45.8% White: 94% Excluded participants with previous heart failure	121,245	Median 8.9 years	Incident heart failure
Jawad et al. (2024)	Mean age: 56.5 ± 8.1 years Male: 45.9% Mean body mass index: 27.4 ± 4.8 kg/m^2^ White: 95% Excluded participants with previous heart failure or missing covariates	271,794	Median 13.7 years	Incident heart failure
Jawad et al. (2024)	Mean age: 61.7 ± 6.3 years Male: 78.6% Mean body mass index: 30.2 ± 5.7 kg/m^2^ White: 95% Included participants with heart failure	1239	Median 13.7 years	CVD mortality
Lu et al. (2024)	Mean age: ~55.4 ± 8.1 years Male: 43.8% White: 95% Excluded participants with hypertension or on antihypertensive medications	195,250	Mean 12.48 years	Incident hypertension
Schuchardt et al. (2024)	Mean age: 56.2 ± 8.1 years Male: 45.5% Mean body mass index: 27.1 ± 4.5 kg/m^2^ White: 96% Excluded participants with type 1 or type 2 diabetes, gestational diabetes, HbA1c‐defined type 2 diabetes mellitus and those with missing covariates	92,762	No follow‐up: cross‐sectional study	Prevalence of prediabetes
Zhuang et al. (2022)	Mean age: ~55.7 ± 8.1 years Male: 44.5% Mean body mass index: ~27.2 ± 4.5 kg/m^2^ White: 95% Excluded participants with CVD, cancer, or diabetes; with incident T2D that occurred within the first 2 years of follow‐up; with baseline HbA1c level ≥ 48 mmol/mol or fasting glucose level ≥ 11.1 mmol/L; with extreme BMI (< 18.5 or > 40 kg/m^2^); or with missing data	92,854	Mean 11.6 years	Incident T2D
Tian et al. (2025)	Median age: 60 (54–63) years Male: 60.3% Median body mass index: 30.7 (27.3–34.8) kg/m^2^ White: 88% Included participants with T2D. Excluded participants with incomplete dietary information, who withdrew from study, or with pre‐existing macrovascular complications, microvascular complications or cancer	20,338	Median 13.2 years	Macrovascular complications with T2D: Composite Incident CHD Incident peripheral artery disease Incident stroke Incident heart failure Microvascular complications with T2D: Composite Incident diabetic neuropathy Incident diabetic kidney disease Incident diabetic retinopathy
Zhuang et al. (2024)	Mean age: ~56.5 ± 8.1 years Male: 45.6% Mean body mass index: ~27.4 ± 4.7 kg/m^2^ White: 94% Excluded participants with NAFLD or other liver diseases or who had histories of drug or alcohol abuse and those who developed severe NAFLD within the first two years of follow‐up	116,223	Mean 12.3 years	Incident severe NAFLD
Tian et al. (2023)	Mean age: 56.8 ± 7.8 years Male: 47.3% Mean body mass index: 26.9 ± 4.6 kg/m^2^ Excluded participants with atypical diet, unreliable energy intake (< 500 or > 3500 kcal/day for women and < 800 or > 4000 kcal/day for men), incomplete phenotype (nutrients, BMI, income, and education), HBV/HCV infection, liver‐related disease (hemochromatosis, viral hepatitis, Wernicke's disease, and Wilson disease), liver damage drug use, alcohol use or alcoholic diseases	93,399	No follow‐up: cross sectional study	Prevalence of NAFLD

### 3.3 Findings

Table 2 summarizes the findings from the 16 studies included in the review including the statistical tests used, the statistical adjustments made for confounders, the effects described as hazard ratio (HR) or odds ratio (OR) along with 95% confidence interval (CI) and whether the studies showed statistical significance as well as the conclusions made.

**TABLE 2:** Findings of studies included in the systematic review.

Study	Cardiometabolic outcome reported	Statistical test used	Findings	Conclusion
Jin et al. (2023)	Incident CHD	Cox proportional hazards models Model 1 stratified by age and sex; Model 2 stratified by age and sex, and adjusted for ethnicity, education, region, Townsend deprivation index, BMI, smoking, and alcohol; Model 3 further mutually adjusted Model 2 for other fatty acids	Model 2: Total omega‐3 fatty acids: HR 0.99 (95% CI: 0.95,1.02) DHA: HR 0.94 (95% CI: 0.91,0.98) Model 3: Total omega‐3 fatty acids: HR 0.91 (95% CI: 0.87, 0.94; *p* < 0.001) DHA: HR 0.92 (95% CI: 0.88, 0.95; *p* < 0.001)	Plasma omega‐3 fatty acids (total, DHA) are associated with lower risk of CHD
Zhuang et al. (2023)	Incident CHD	Cox multivariable regression models Model 1 adjusted for age and sex; Model 2 was further adjusted for race, assessment center, education, Townsend deprivation index, household income, smoking, alcohol consumption, physical activity, history of hypertension, history of high cholesterol, history of diabetes, family history of CVD, vitamin supplement use, mineral supplement use, and aspirin use; Model 3 was further adjusted for BMI; Model 4 was further adjusted for remaining plasma fatty acids	Q4 vs. Q1 of Model 4 Plasma total omega‐3 fatty acids: HR 0.84 (95% CI: 0.75, 0.95; *P* trend = 0.004) DHA: HR 0.94 (95% CI: 0.89, 1.00; *P* trend = 0.003) Non‐DHA omega‐3 fatty acids: HR 1.00 (95% CI: 0.95, 1.04)	Plasma omega‐3 fatty acids (total, DHA) are associated with lower risk of CHD
Fan et al. (2023)	CHD mortality	Multivariable linear regression; Logistic regression Adjusted for age, sex, ethnicity, education, alcohol status, smoking status, physical activity, Townsend deprivation index, medication use, and baseline disease status	DHA: OR 0.88 (95% CI: 0.81, 0.96; *p* = 0.004)	Plasma DHA is associated with lower risk of CHD mortality
Zhang et al. (2024)	CVD mortality	Multivariable Cox proportional hazards regression Adjusted for age, sex, race, Townsend deprivation index, assessment centre, BMI, smoking status, alcohol intake, physical activity, comorbidities	Q5 vs. Q1 Total omega‐3 fatty acids: HR 0.68 (95% CI: 0.57, 0.82; *P* trend < 0.001)	Plasma total omega‐3 fatty acids are associated with lower CVD mortality
O'Keefe et al. (2024)	CVD mortality	Cox proportional hazard models Adjusted for age, sex, ethnicity, marital status, employment status, education, Townsend deprivation index, physical activity, smoking status, BMI, prevalent dyslipidemia, prevalent hypertension, prevalent diabetes, physical activity, self‐rated health, total plasma omega‐6 fatty acids	Q5 vs. Q1 DHA: HR 0.79 (95% CI: 0.73, 0.87; *P* trend < 0.001)	Plasma DHA is associated with lower risk of CVD mortality
Zhang et al. (2025)	Incidence of new onset major adverse cardiovascular events	Cox proportional hazards models Adjusted for age, sex, ethnicity, education, average household income, smoking status, alcohol consumption frequency, weekly minutes of moderate physical activity, total fruit intake, total vegetable intake, systolic and diastolic blood pressure, use of antihypertensive medication, insulin use, cholesterol‐lowering medication use, family history of CVD, presence of T2D, presence of hypertension	Total omega‐3 fatty acids: Haemorrhagic stroke: HR 0.80 (95% CI: 0.73, 0.89; *p* < 0.05) Cardiovascular mortality: HR 0.71 (95% CI: 0.68, 0.75; *p* < 0.05) DHA: Haemorrhagic stroke: HR 0.85 (95% CI: 0.76, 0.94; *p* < 0.05) Ischaemic stroke: HR 0.85 (95% CI: 0.79, 0.90; *p* < 0.05)	Plasma omega‐3 fatty acids (total, DHA) are associated with lower risk of major adverse cardiovascular events
Ren et al. (2025)	Incidence of major adverse cardiovascular events in middle‐aged and elderly participants	Multivariable adjusted Cox proportional hazards models Model 1 adjusted for age, gender, ethnicity, Townsend deprivation index, BMI, alcohol consumption, smoking status, physical activity, sleep duration, eGFR, systolic and diastolic blood pressure, HbA1c, fasting time, and medication status (blood pressure medication and diabetes medication); Model 2 further adjusted for LDL‐cholesterol, HDL‐cholesterol, and triglycerides; Model 3 was adjusted similarly to Model 1 but further incorporated metabolic biomarkers	Total omega‐3 fatty acids in middle‐aged: HR 0.99 (95% CI: 0.95, 1.04; *p* = 0.802) DHA in middle aged: HR 0.97 (95% CI: 0.92, 1.02; *p* = 0.235) Total omega‐3 fatty acids in elderly: HR 0.95 (95% CI: 0.91, 0.99; *p* = 0.07) DHA in elderly: HR 0.94 (95% CI: 0.90–0.98; *p* = 0.01)	Plasma DHA is associated with lower risk of major adverse cardiovascular events in elderly but not in middle aged
Liu, Ao, et al. (2025)	CVD mortality	Cox proportional hazard regression models Model 1 adjusted for age and sex; Model 2 further adjusted for BMI, race, education, assessment center, smoking status, alcohol consumption, Townsend deprivation index, household income, physical activity, history of hypertension, high cholesterol, cardiovascular disease, diabetes, and cancer, the use of aspirin, vitamin supplement, and mineral supplement; Model 3 was further adjusted for other plasma fatty acids	Q4 vs. Q1 Total omega‐3 fatty acids: OR 0.63 (95% CI: 0.55, 0.72; *P* trend < 0.001)	Plasma total omega‐3 fatty acids are associated with lower risk of CVD mortality
Ma et al. (2023)	Incidence of heart failure	Cox proportional hazards regression Adjusted for age, sex, ethnicity, BMI, physical activity, smoking, drinking alcohol, diabetes, hypertension, Townsend deprivation index, and family history of CVD	Total omega‐3 fatty acids: HR 0.47 (95% CI: 0.36, 0.61; *p* < 0.001) DHA: HR 0.53 (95% CI: 0.39, 0.72; *p* < 0.001)	Plasma omega‐3 fatty acids (total, DHA) are associated with lower risk of heart failure
Jawad et al. (2024)	Incident heart failure	Cox proportional hazard models Adjusted for age, sex, ethnicity, education, smoking, alcohol consumption, self‐reported exercise, BMI, waist circumference, CHD, use of cholesterol‐lowering medication, low‐density lipoprotein to high‐density lipoprotein cholesterol ratio, prevalent hypertension, prevalent T2D	Total omega‐3 fatty acids: HR 0.82 (95% CI, 0.77, 0.86; *p* < 0.001) DHA: HR 0.89 (95% CI: 0.84, 0.94; *p* < 0.001) Non‐DHA omega‐3 fatty acids: HR 0.79 (95% CI: 0.75, 0.84; *p* < 0.001)	Plasma omega‐3 fatty acids (total, DHA, non‐DHA) are associated with lower risk of heart failure
Jawad et al. (2024)	CVD mortality in those with heart failure	Cox proportional hazard models As above	Total omega‐3 fatty acids: HR 0.57 (95% CI: 0.39, 0.83; *p =* 0.02) DHA: HR 0.60 (95% CI: 0.42, 0.86; *p* = 0.01) Non‐DHA omega‐3 fatty acids: HR 0.62 (95% CI: 0.44, 0.89; *p =* 0.003)	Plasma omega‐3 fatty acids (total, DHA, non‐DHA) are associated with lower risk of CVD death in those with heart failure
Lu et al. (2024)	Incidence of hypertension	Cox proportional hazards model Model 1 adjusted for age and sex; Model 2 further adjusted for other confounding factors according to previous studies, including race, Townsend deprivation index, physical activity, BMI, alcohol consumption, smoking status, diet score, diabetes status and family history of disease	Total omega‐3 fatty acids Model 1: HR 0.93 (95% CI: 0.92, 0.94; *P* trend = 0.002) Model 2: HR 0.984 (95% CI, 0.973, 0.995; *P* trend = 0.439)	Model 1: omega‐3 fatty acids associated with lower risk of hypertension Model 2: No association
Schuchardt et al. (2024)	Prevalence of prediabetes	Logistic regression models Model 1adjusted for age, sex, ethnicity, BMI, and waist circumference; Model 2 further adjusted for smoking, alcohol consumption, Townsend deprivation index, education, physical activity, urbanicity, polygenic risk score, familial T2DM, use of cholesterol‐lowering drugs, steroids, blood pressure medications, hormone replacement, and use of oral contraceptives	Q5 vs. Q1 Total omega‐3 fatty acids: OR 0.77 (95% CI: 0.72, 0.82; *p* < 0.001) DHA: OR 0.62 (95% CI: 0.58, 0.67; *p* < 0.001) Non‐DHA omega‐3 fatty acids: OR 0.91 (95% CI: 0.85, 0.97; *p* < 0.05)	Plasma omega‐3 fatty acids (total, DHA, non‐DHA) are associated with lower risk of prediabetes
Zhuang et al. (2022)	Incidence of T2D	Multivariable Cox proportional hazards regression Model 1 adjusted for age and sex; Model 2 further adjusted for assessment centre, ethnicity, BMI, Townsend deprivation index, education, annual household income, physical activity, smoking, alcohol consumption, history of hypertension, history of hypercholesteremia, family history of diabetes, and use of mineral supplements, vitamin supplements, and aspirin; Model 3 was further adjusted for remaining plasma fatty acids	Risk of incident T2D with Model 3: Total omega‐3 fatty acids: HR 0.90 (95% CI: 0.85, 0.95; *P* trend < 0.001) DHA: HR 0.93 (95% CI: 0.87, 1.00; *P* trend < 0.001) Non‐DHA omega‐3 fatty acids: HR 0.90 (95% CI: 0.85, 0.96*; P* trend < 0.001)	Plasma omega‐3 fatty acids (total, DHA, non‐DHA) are associated with lower risk of developing T2D
Tian et al. (2025)	Incidence of macrovascular and microvascular complications with T2D	Multivariable Cox proportional‐hazards regression model Model 1 adjusted for age and sex; Model 2 further adjusted for Townsend deprivation index, ethnicity group, educational attainment, smoking status, alcohol consumption, total physical activity, fruits, vegetables, vegetable oil, red meat, poultry, processed meat, oily fish, nonoily fish, BMI, diabetes duration, hypertension, HbA1c, antidiabetic medication, antihypertensive medication, lipid‐lowing medication, aspirin, vitamin supplementation, mineral and other dietary supplementation; Fasting time was additionally adjusted in the analysis of omega‐3 fatty acids.	Model 2 Q4 vs. Q1: Total omega‐3 fatty acids: Composite macrovascular complications: HR 0.81 (95% CI: 0.69, 0.96; *P* trend < 0.028) CHD: HR 0.76 (95% CI: 0.62, 0.93; *P* trend < 0.014) Heart failure: HR 0.93 (95% CI: 0.69, 1.26; *P* trend < 0.728) Peripheral artery disease: HR 0.76 (95% CI: 0.50, 1.14; *P* trend < 0.237) Stroke: HR 1.07 (95% CI: 0.72, 1.60; *P* trend < 0.563) Composite microvascular complications: HR 0.90 (95% CI: 0.75, 1.07; *P* trend < 0.203) Diabetic neuropathy: HR 0.65 (95% CI: 0.43, 0.99; *P* trend < 0.067) Diabetic kidney disease: HR 0.96 (95% CI: 0.76, 1.22; *P* trend < 0.451) Diabetic retinopathy: HR 0.89 (95% CI: 0.69, 1.14; *P* trend < 0.298) DHA: Composite macrovascular complications: HR 0.68 (95% CI: 0.57, 0.81; *P* trend < 0.001) CHD: HR 0.63 (95% CI: 0.51, 0.77; *P* trend < 0.001) Peripheral artery disease: HR 85 (95% CI: 0.56, 1.28; *P* trend < 0.217) Stroke: HR 1.13 (95% CI: 0.76, 1.68; *P* trend < 0.798) Composite microvascular complications: HR 0.85 (95% CI: 0.71, 1.01; *P* trend 0.014) Diabetic kidney disease: HR 0.92 (95% CI: 0.72, 1.17; *P* trend < 0.200) Diabetic retinopathy: HR 0.81 (95% CI: 0.63, 1.04; *P* trend < 0.055)	Plasma total omega‐3 fatty acids and DHA are associated with lower risk of macrovascular complications and CHD in people with T2D; plasma DHA is associated with lower risk of microvascular complications in people with T2D
Zhuang et al. (2024)	Incidence of severe non‐alcoholic fatty liver disease	Cox proportional‐hazard models Model 1 adjusted for age and sex; Model 2 was further adjusted for ethnicity, assessment center, BMI, education, household income, smoking status, alcohol consumption, physical activity, history of hypertension, CVD, high cholesterol and cancer, diabetes, aspirin use, vitamin use, and mineral use; Model 3 was further adjusted for remaining fatty acids	Model 3 Q4 vs. Q1 Total omega‐3 fatty acids: HR 0.79 (95% CI: 0.65, 0.97; *P* trend = 0.020) DHA: HR 0.92 (95% CI: 0.71, 1.19; *P* trend = 0.278) Non‐DHA omega‐3 fatty acids: HR 0.91 (95% CI: 0.73, 1.14; *P* trend = 0.339)	Plasma total omega‐3 fatty acids (but not DHA or non‐DHA omega‐3 fatty acids) are associated with lower risk of severe NAFLD
Tian et al. (2023)	Incidence of NAFLD	Multivariate linear regression Model 1 adjusted for age, sex, and BMI; Model 2 further adjusted for sedentary time, exercise, income and education	Total omega‐3 fatty acids: No association found; results not reported. DHA: OR 0.70 (95% CI: 0.59, 0.82; *p* < 0.001)	Plasma DHA is associated with lower risk of NAFLD

First, with regard to CVD‐related outcomes, Jin et al. (2023) reported that there was no association between plasma omega‐3 fatty acids and CHD risk in a fully adjusted model; however, a slight but significant inverse association between plasma DHA and CHD risk was observed. When other fatty acids were also adjusted for, there was a stronger inverse association for both total omega‐3 fatty acids and DHA. Zhuang et al. (2023) reported an inverse association between plasma omega‐3 fatty acids and risk of CHD: total plasma omega‐3 fatty acids and DHA were statistically significant, but non‐DHA omega‐3 fatty acids were not. Both Zhang et al. (2024) and Liu, Ao, et al. (2025) reported an inverse association between total omega‐3 fatty acids and CVD mortality, while O'Keefe et al. (2024) and Fan et al. (2023) reported that higher plasma DHA was associated with significantly lower risk of mortality due to CVD and to CHD, respectively. Zhang et al. (2025) reported that higher omega‐3 fatty acids were associated with lower risks of haemorrhagic stroke and cardiovascular mortality, and that higher plasma DHA was associated with a lower risk of both ischemic and haemorrhagic stroke. Although Ren et al. (2025) found that total omega‐3 fatty acids and DHA had no association with the incidence of major adverse cardiovascular events in middle‐aged individuals, among elderly participants, total omega‐3 fatty acids and DHA were both associated with a reduced risk of major adverse cardiovascular events. Ma et al. (2023) found that plasma omega‐3 fatty acids and DHA were associated with lower risk of heart failure. Likewise, Jawad et al. (2024) found an inverse association between plasma total omega‐3 fatty acids, DHA and non‐DHA omega‐3 fatty acids and the incidence of heart failure. They also found that plasma omega‐3 fatty acids (total, DHA, non‐DHA) were associated with lower risk of CVD death in those with heart failure at baseline (Jawad et al. 2024). Finally, Lu et al. (2024) found contrasting results for the two statistical models they used, where model 1 showed high levels of omega‐3 fatty acids were inversely associated with risk of hypertension, but the further adjusted model 2 showed no significant effect.

Secondly, in relation to metabolic disease‐related outcomes, Schuchardt et al. (2024) reported an inverse association between plasma omega‐3 fatty acids and prevalence of prediabetes in a cross‐sectional investigation. Consistent with this, Zhuang et al. (2022) reported a 10% lower T2D risk in individuals with higher plasma omega‐3 fatty acids. Tian et al. (2025) found that higher plasma omega‐3 fatty acids were associated with lower risks of various macrovascular and microvascular complications of T2D (see Table 2 for details). However, some conditions such as heart failure, peripheral artery disease, stroke, composite microvascular complications, diabetic kidney disease, and diabetic retinopathy did not show a statistically significant relationship, providing no evidence to support that high omega‐3 fatty acids, especially DHA, lower their incidence risk. Tian et al. (2023) reported that higher plasma DHA was inversely associated with prevalence of NAFLD; however, no association between total omega‐3 fatty acids and NAFLD was found. Zhuang et al. (2024) found a 21% reduction of risk of severe NAFLD with higher plasma omega‐3 fatty acids.

### 3.4 Assessment of Study Quality

The findings of study quality assessment are shown in Tables 3 and 4. All 14 prospective cohort studies scored 8 out of 9, losing one point because the studied cohort is not fully representative of the desired population. The two cross‐sectional studies scored 7 out of 9, losing one point because the studied cohort is not fully representative of the desired population and another because the fatty acids were assessed by an acceptable method (NMR spectroscopy) but not the gold standard method (gas chromatography or gas chromatography–mass spectrometry (Brenna et al. 2018)).

**TABLE 3:** Summary of study quality assessment using the Newcastle‐Ottawa Scale (prospective cohort studies).

Study	Selection	Comparability	Outcome	Overall score/9
Jin et al. (2023)	3	2	3	8
Zhuang et al. (2023)	3	2	3	8
Fan et al. (2023)	3	2	3	8
Zhang et al. (2024)	3	2	3	8
O'Keefe et al. (2024)	3	2	3	8
Zhang et al. (2025)	3	2	3	8
Ren et al. (2025)	3	2	3	8
Liu, Ao, et al. (2025)	3	2	3	8
Ma et al. (2023)	3	2	3	8
Jawad et al. (2024)	3	2	3	8
Lu et al. (2024)	3	2	3	8
Zhuang et al. (2022)	3	2	3	8
Tian et al. (2025)	3	2	3	8
Zhuang et al. (2024)	3	2	3	8

**TABLE 4:** Summary of study quality assessment using the adapted Newcastle‐Ottawa Scale (cross‐sectional studies).

	Study sample selection	Assessment of exposure and outcome	Confounding factors	Overall score/9
Schuchardt et al. (2024)	1	3	3	7
Tian et al. (2023)	1	3	3	7

## 4 Discussion

The long chain, highly unsaturated omega‐3 fatty acids EPA and DHA are crucial for optimal cellular function, resilient physiology and maintenance of health (Djuricic and Calder 2023, 2025). Both EPA and DHA serve several roles in the body, ranging from being anti‐inflammatory to acting as triglyceride‐lowering agents (Harris 2001; Calder 2020; Choi and Calder 2024). Research to date has highlighted their potential benefits for cardiometabolic health, especially their association with reduced risk of CHD incidence and mortality (Chowdhury et al. 2014; Del Gobbo et al. 2016; Harris et al. 2021) but also T2D incidence (Qian et al. 2021). Despite such evidence, inconsistencies in the literature and the blurring of the roles of EPA and DHA in prevention and treatment of cardiometabolic diseases (Toth et al. 2022) call for continued efforts to confirm that these fatty acids have a role in cardiometabolic disease prevention. UKBB with its great number of well‐phenotyped participants and its fairly long period of follow‐up offers the opportunity to study the association of omega‐3 fatty acids with cardiometabolic health outcomes. In UKBB case ascertainment is from a variety of common sources, including hospital admission records, diagnosis using ICD10 and ICD9 codes, procedural codes, primary care records using diagnostic codes such as Read codes or Snomed codes, and prescription and death registry data. For most, if not all, outcomes described in the included studies there will be ICD10 coding data (e.g., for MI, CVD etc.) and there may also be a diagnosis from primary care records or from disease registries. The strength of this linkage‐based approach using multiple sources for identification of a case is that it increases both sensitivity and specificity.

In UKBB total omega‐3 fatty acids, DHA and what is referred to as “non‐DHA omega‐3 fatty acids” have been measured in plasma using NMR spectroscopy. This has enabled associations between plasma omega‐3 fatty acids and measures of cardiometabolic health to be made cross‐sectionally at baseline (*n* = 2 studies) and longitudinally as a prospective cohort with follow‐up of between 11 and 14 years (*n* = 14 studies). Findings were categorized into 2 main outcomes: cardiovascular (*n* = 11 studies) or metabolic (*n* = 5 studies). The number of participants ranged from 20,338 to 273,834 across the studies (except for one small study evaluating CVD mortality in participants with pre‐existing heart failure). All studies adjusted their findings for multiple confounding factors, increasing the rigor of this dataset. All 16 studies found protective and beneficial associations between plasma total omega‐3 fatty acids and/or DHA and cardiometabolic health; no study identified any harmful association. Thus, having a higher concentration of total omega‐3 fatty acids or DHA in blood was associated with a lower risk of multiple cardiometabolic disease outcomes including risk of developing CHD, heart failure, T2D and NAFLD and risk of mortality from CVD. We did not conduct a meta‐analysis of this dataset because all studies were derived from the same source (i.e., UKBB) and therefore are not statistically independent.

The findings of this systematic review support other analyses of the literature. For example, a meta‐analysis of cohort studies reported that those in the highest tertile of dietary intake of EPA + DHA or of circulating concentrations of EPA, DHA or EPA + DHA had a lower risk of “coronary outcomes” than those in the lowest tertile (Chowdhury et al. 2014). Studies that have pooled data from multiple cohort studies established independent associations between higher EPA or DHA status (e.g., in blood) and lower risk of mortality from CHD (Del Gobbo et al. 2016) and CVD (Harris et al. 2021). More recently, combining data from 3 cohort studies including UKBB revealed a significant inverse association between plasma total omega‐3 fatty acids and plasma DHA and risk of CHD in the follow up period (Shi et al. 2025). Taking the consistent findings from meta‐analyses and pooling studies that bring together findings from multiple different cohorts and the current research collating multiple findings from a single large cohort supports the view that omega‐3 fatty acids are associated with improved cardiometabolic health and reduced risk and severity of cardiometabolic disease. The broad effects of omega‐3 fatty acids are likely due to their ability to impact a wide range of relevant cells, tissues, physiological processes and risk factors (AbuMweis et al. 2018; Calder 2018; Innes and Calder 2020). This view is also supported by research showing that intervention with EPA and DHA reduces atherosclerotic plaque burden and the prevalence of high‐risk plaques (Feuchtner et al. 2021) and enhances plaque stability (Thies et al. 2003; Cawood et al. 2010).

The studies from UKBB included in the current systematic review reveal that the associations of outcomes with total plasma omega‐3 fatty acids are often shared with those with plasma DHA, but rarely with non‐DHA omega‐3 fatty acids. This suggests that DHA may have a more important role in maintaining cardiometabolic health than other omega‐3 fatty acids. The latter will include EPA and docosapentaenoic acid (DPA). However, this distinction has been difficult to tease out in human intervention trials which have been conducted mostly using combinations of EPA and DHA, with limited research on DPA (Ghasemi Fard et al. 2021). Nevertheless, a small number of randomized controlled trials have compared the effect of near pure EPA with those of near pure DHA given at the same dose. A systematic review of 24 publications from 9 such trials conducted in generally healthy participants or in those with dyslipidaemias indicated that both EPA and DHA beneficially modify a number of cardiometabolic risk factors but that often the effects of DHA are greater, although the difference seems to be generally modest (Choi and Calder 2024). This conclusion contrasts with findings from some large randomized controlled trials in patients with existing CVD or at high risk, where EPA monotherapy had benefits (Yokoyama et al. 2007; Bhatt et al. 2019) but the combination of EPA and DHA did not (Nicholls et al. 2020; Kalstad et al. 2021), as discussed elsewhere (Sharma et al. 2020; Calder and Deckelbaum 2021; Sweeney et al. 2023). Nevertheless, there may be a distinction between the roles of EPA and DHA in cardiometabolic disease prevention and in its treatment. This needs further research.

The current analysis based on publications from UKBB demonstrates an association between having higher omega‐3 fatty acids, especially DHA, in the bloodstream and lower risk of adverse cardiometabolic outcomes. Seafood, especially oily fish, is the only rich dietary source of EPA and DHA. “Fish oil” supplements are also sources of EPA and DHA. In general, those eating more fish (Harris et al. 2007; Sands et al. 2005; Sharman et al. 2025) or using fish oil supplements (Browning et al. 2012; Harris et al. 2007; Rees et al. 2006; Sharman et al. 2025; Yaqoob et al. 2000) have higher blood EPA, DHA, total omega‐3 fatty acids, and non‐DHA omega‐3 fatty acids. In UKBB, eating fish was associated with a lower risk of CVD (Zhang et al. 2021). UKBB includes a question on the use of fish oil supplements with a dichotomous answer yes or no and no further detail, and a number of studies have been published investigating the association between fish oil use and health outcomes. Recently, Abuknesha et al. (2025) reviewed 34 UKBB publications examining fish oil supplement use and incident disease. Across the included studies, 56 fish oil supplement use–“disease” relationships were reported. Of these 56 relationships, 44 were statistically significant, and of those 44, 43 were favorably related to fish oil use, including many cardiometabolic outcomes such as incident CHD and death from CVD. Taken together, the UKBB findings suggest that higher fish consumption, higher dietary intake of EPA and DHA, greater use of fish oil supplements, and having higher blood total omega‐3 fatty acids and DHA, which are all inter‐related, are each associated with better cardiometabolic health and lower future disease risk.

There is currently significant interest in omega‐3 fatty acids and risk of atrial fibrillation (Fatkin et al. 2022; Herrmann and Herrmann 2024; O'Keefe, O'Keefe, Abuissa, et al. 2025; Qian et al. 2023). We did not identify any papers from UKBB investigating the associations between plasma omega‐3 fatty acids and risk of atrial fibrillation. Our literature search was restricted to publications up to September 2025. More recently, one such paper has been published (O'Keefe, O'Keefe, Tintle, et al. 2025). Using data from just over 261,000 individuals followed up for an average of 12.7 years after controlling for multiple confounders, the risk of atrial fibrillation was decreased with increasing total omega‐3 fatty acids, DHA, and non‐DHA omega‐3 fatty acids.

The current systematic review has a number of strengths. It brings together findings from publications based on UKBB which has a large sample size (over 500,000 participants) with studies included in the review having a high number of participants (20,338 to 273,834). Furthermore, the follow‐up period was reasonably long, typically between 11 and 14 years. Studies excluded those participants already exhibiting the outcome of interest and in some cases excluded those who displayed the outcome early in follow‐up on the assumption that it may have already been present at study entry but not yet diagnosed or evident. UKBB participants are extremely well phenotyped and another strength of this review is that the risk of confounding bias is reduced in all included studies due to the adjustments for multiple confounders and covariates. Another strength is that the health‐related outcomes were gathered by linking UKBB with UK medical records, increasing the rigor of assessing the outcomes.

There are however some limitations. Despite the large data pool, the age range of participants in UKBB at study entry was 40–69 years, which means it is difficult to apply the results to individuals outside this range (younger or older). Also, the follow‐up period may not be long enough for the manifestation of disease, especially for those recruited at a younger age, as disease development may be in later years. Another limitation of UKBB, and hence of publications from it, is that it does not reflect the current ethnic diversity of the UK population: 94.6% of the participants in UKBB were of white Caucasian descent. While this was representative of UK demographics when UKBB started, currently only 81.7% of the UK population are of white Caucasian descent (Office for National Statistics 2022). Furthermore, UKBB recruited individuals who were wealthier and healthier than the UK average. Of note, recruits to UKBB represent about 5.4% of those invited. Thus, UKBB participants are not truly representative of the total UK adult population in terms of age, ethnicity, wealth and health and the results cannot be generalized to the entire UK population, perhaps even to the entire middle‐aged to late middle‐aged population. It is also worth noting that NMR spectroscopy, as used in UKBB, is not a gold standard methodology to measure plasma fatty acids and it does not explicitly measure EPA, unlike the gold standards gas chromatography and gas chromatography–mass spectrometry. This is a further limitation of UKBB. It is also important to highlight that the omega‐3 fatty acids were measured in plasma where their concentrations may be modified by eating a meal; in comparison, red blood cells are stable to this and so are considered to better reflect habitual or long‐term omega‐3 fatty acid intake. Nevertheless, well conducted intervention trials show that in the absence of any dietary change neither EPA nor DHA change in fasting plasma or serum lipids over at least one year (Browning et al. 2012; Katan et al. 1997).

It is important to note that the studies included in this review report associations and cannot demonstrate a causal link between the exposure (plasma omega‐3 fatty acids) and the outcomes. Although extensive statistical adjustments were made in all studies, there could still be residual confounding. Given that those who eat fish or take omega‐3 supplements are likely to have higher plasma omega‐3 fatty acid levels, it is possible that having higher plasma omega‐3 fatty acid status is simply a marker for a more healthy diet or, even more generally, a more healthy lifestyle. However to help mitigate against this, most of the included studies controlled for smoking, alcohol consumption and physical activity and many controlled for different aspects of diet such as fruit and vegetable consumption, meat consumption, diet score or use of vitamin and mineral supplements (see Table 2). This suggests that the findings reported in the included studies do relate to omega‐3 fatty acids and not to a more healthy diet or more healthy lifestyle.

The findings from the UKBB publications included here demonstrate that having higher concentrations of omega‐3 fatty acids in the blood, especially DHA, has a beneficial association to cardiometabolic health. The main sources of these fatty acids are seafood, especially oily fish, and supplements. On average in the UK, individuals are consuming far less oily fish and EPA and DHA than recommended by the Government (Lewis et al. 2025). The current review indicates that there may be a health cost to this. More education of the benefits of fish and omega‐3 fatty acids is required in order to improve population health.

In conclusion, this systematic review of publications arising from UKBB consistently demonstrates a significant association between higher total omega‐3 fatty acids and DHA in blood and lower risk of common adverse cardiometabolic outcomes including risk of CHD, T2D, fatty liver disease, and CVD mortality. This research provides additional support for guidance for the population to achieve recommended intakes of these fatty acids.

## Author Contributions

P.C.C. conceptualized the research; W.T.F. and P.C.C. designed the search strategy; W.T.F. conducted the literature searching and study selection – each paper was discussed with P.C.C. to ensure agreement on inclusion; W.T.F. extracted the data and collated this onto tables (study characteristics, study findings), and P.C.C. checked each entry for accuracy against the original publication with any discrepancies discussed to ensure agreement; P.C.C. conducted the assessment of study quality; W.T.F. wrote the draft of the manuscript; P.C.C. reviewed the draft; W.T.F. and P.C.C. read, critically reviewed, and approved the final manuscript.

## Funding

No funding was associated with this research.

## Conflicts of Interest

W.T.F. has no interests to declare. P.C.C. acts as an ad hoc consultant/advisor to BASF, Bayer Consumer Care, Danone Nutricia Research, dsm‐firmenich, Bunge, Fresenius‐Kabi, B. Braun Melsungen, Nestle, Baxter Healthcare, Abbott Nutrition, Haleon, Holland and Barrett, Royal Cosun, AAK and Natures Crops; has received speaking honoraria from dsm‐firmenich, Fresenius‐Kabi, Abbott Nutrition, and Eqology; and has recently received study products from dsm‐firmenich.

## Supporting information


**Table S1:** Search string used for identifying publications.

## Data Availability

Data sharing not applicable to this article as no datasets were generated or analyzed during the current study.
