# Supplementary material for: Circulating Omega‐3 Fatty Acids and Cardiometabolic Outcomes: A Systematic Review of Publications From UK Biobank
Source: Lipids. 2026 Jul 29;61(6):783–801. doi: 10.1002/lipd.70076 (PMC13751176; doi:10.1002/lipd.70076)
Supplement: Supplementary file 1 [file LIPD-61-783-s001.docx]

**Supplementary Table 1. Search string used for identifying publications.**

| 1 | (omega 3 or omega-3) |
| --- | --- |
| 2 | (UK Biobank or UK BB) |
| 3 | (PUFAs or polyunsaturated fatty acids) |
| 4 | (EPA or eicosapentaenoic acid) |
| 5 | (DHA or docosahexaenoic acid) |
| 6 | 1 or 3 or 4 or 5 |
| 7 | 2 and 6 |
